# Milk Modulates the Gastrointestinal Stability of Tick‐Borne Encephalitis Virus: Implications for Alimentary Transmission

**DOI:** 10.1002/jmv.70778

**Published:** 2025-12-24

**Authors:** Martin Machacek, Michaela Berankova, Jiri Salat, Daniel Ruzek

**Affiliations:** ^1^ Department of Experimental Biology, Faculty of Science Masaryk University Brno Czechia; ^2^ Laboratory of Emerging Viral Infections Veterinary Research Institute Brno Czechia; ^3^ Laboratory of Arbovirology, Institute of Parasitology Biology Centre of the Czech Academy of Sciences Ceske Budejovice Czechia

## Abstract

Tick‐borne encephalitis virus (TBEV) can be transmitted alimentarily through contaminated dairy products, yet the mechanisms by which the virus survives the digestive tract remain poorly understood. In this study, we investigated the stability of TBEV in milk under simulated gastrointestinal conditions. While milk is known to preserve viral infectivity at low temperatures, our results demonstrate that in the gastric environment and at physiological temperature, it exerts a destabilizing effect, significantly reducing TBEV viability. All major milk fractions—whey, casein, and lipids—contribute to this effect. This highlights the necessity for rapid transit of virus‐containing milk through the stomach to avoid inactivation. Conversely, in the intestinal environment, milk protects TBEV from bile salt‐mediated inactivation, allowing viral persistence in the upper small intestine. Casein was identified as the primary protective component counteracting bile salt disruption. These findings offer new insights into how milk can simultaneously act as a transmission vehicle and modulator of TBEV stability, advancing our understanding of alimentary infection routes and their implications for public health.

## 1 Introduction

Tick‐borne encephalitis virus (TBEV; *Orthoflavivirus encephalitidis*) is an enveloped, positive‐sense single‐stranded RNA virus belonging to the *Orthoflavivirus* genus within the *Flaviviridae* family [1, 2]. It is the causative agent of tick‐borne encephalitis (TBE), a viral disease that primarily affects the central nervous system in humans and is endemic to large parts of Europe and Asia [1].

While the primary route of TBEV transmission to humans is through the bite of infected *Ixodes* ticks, an alternative transmission pathway through the alimentary route—specifically, the consumption of unpasteurized milk and dairy products from sheep, goats, or cows—has been well documented and remains a public health concern in endemic areas [3, 4, 5]. The first known alimentary outbreak of TBEV occurred in 1951 in Rožňava, Czechoslovakia, linked to the ingestion of contaminated goat milk [6]. Since then, multiple outbreaks have been reported in various countries, including Hungary (2011) [7], Croatia (2019) [8], and France (2020) [9], with the majority of alimentary cases being reported in Slovakia, where incidence has shown a rising trend in recent years [4, 10].

Infected milk and dairy products from goats, sheep, and cows represent a recognized risk of alimentary TBEV transmission. Pasteurization has been shown to inactivate the virus effectively [11], mitigating the risk. One unconfirmed historical report also suggested possible TBEV transmission through human breast milk [12], although this has not been corroborated by further studies [13].

Although alimentary infection is not the main transmission route, it is epidemiologically important in rural areas where raw dairy consumption is common. Slovakia reports the highest incidence of alimentary TBE in Europe, accounting for up to 17% of national cases. In contrast, only about 0.9% of TBE cases in the Czech Republic are attributed to this route [14]. The mechanisms underlying TBEV infection via the gastrointestinal (GI) tract are not fully elucidated. Experimental studies have shown that TBEV can replicate in Caco‐2 cells—an established model of human intestinal epithelium [15]. However, conflicting findings exist regarding the virus's stability in the acidic gastric environment. Earlier studies reported TBEV persistence in gastric fluid for up to 2 h even at very low pH (1.49–1.80) [16], whereas more recent in vitro experiments demonstrated complete viral inactivation at pH 2.0 after just 20 min [17]. This discrepancy supports the hypothesis that TBEV likely bypasses gastric degradation under specific conditions and gains access to the host via intestinal epithelium [18].

TBEV exhibits remarkable stability in milk, particularly at refrigeration temperatures (4°C–8°C), with no significant difference in viral stability between milk and cell culture media such as Dulbecco's Modified Eagle Medium (DMEM) or phosphate‐buffered saline (PBS) [19, 20]. However, at body temperature (37°C), viral stability is significantly reduced in milk compared to laboratory media [19], possibly due to enzymatic and compositional factors.

The physiological environment of the GI tract plays a critical role in the success of alimentary infection. Gastric pH is typically between 1.4 and 2.1 in a fasted state but can rise to near‐neutral levels (6–7) shortly after feeding, remaining elevated for up to 2 h [21, 22]. In the duodenum, pH ranges from ~5.0–6.0 in the fed state and 6.5 in the fasted state [23]. Similarly, bile acid concentrations vary from 0.1 mmol/L in the stomach to 12–16 mmol/L in the fed small intestine [24, 25]. These pH shifts and bile acid concentrations likely influence viral stability, protein digestion, and ultimately mucosal absorption.

In terms of gastric transit, caloric content influences gastric emptying time. For example, 500 mL of water exits the stomach within 30–60 min, whereas an equivalent volume of milk may remain for over 2 h [26, 27]. During digestion, milk proteins undergo enzymatic cleavage; casein, which comprises around 80% of milk protein, is rapidly digested by pepsin and is no longer detectable after 20 min in the stomach [28]. Other proteins vary in resistance—lactoferrin is quickly cleaved [29], while β‐lactoglobulins are notably resistant to gastric proteolysis [30, 31].

In this study, we explored the mechanisms by which TBEV may survive and pass through the human GI tract during alimentary infection. Specifically, we examined the virus's stability in milk and its behavior under simulated gastric and intestinal conditions. The study also investigates how different milk fractions affect TBEV stability in the acidic stomach environment and how milk—particularly casein—can protect the virus against bile salts in the intestine. These findings help to clarify how TBEV may remain infectious after oral ingestion, contributing to our understanding of alimentary transmission pathways.

## 2 Materials and Methods

### 2.1 Cells and Virus

A549 cells (ATCC CCL‐185) were maintained in DMEM supplemented with 10% fetal bovine serum (FBS), 100 μg/mL penicillin, 100 μg/mL streptomycin, and 1% l‐glutamine. Cultures were incubated at 37°C in a humidified atmosphere containing 5% CO_2_.

The low‐passage TBEV strain Hypr was obtained from the Collection of Arboviruses at the Institute of Parasitology, Biology Centre of the Czech Academy of Sciences (České Budějovice, Czech Republic). This strain was originally isolated in 1953 from the blood of a 10‐year‐old patient in Brno, Czechoslovakia [32].

### 2.2 Processing of Milk Samples

Commercial cow milk (pasteurized) sourced from Czech producers was bought at a local grocery store. Unpasteurized cow and goat milk were sourced from South Moravian producers, while sheep milk came from a producer in South Bohemia. Pasteurization of goat milk and unpasteurized cow milk from local producer was performed by heating the milk to 63°C for 30 min in a water bath. Pasteurized sheep milk was obtained from a local grocery store. Milk used in experiments was not stored for longer than 2 weeks and its pH was always checked right before conducting the experiment.

Milk samples were fractionated into fat and aqueous components (skim milk) by centrifugation (4000*g*, 30 min, 5°C) as described in Blans et al. [33]. The aqueous phase was further separated into casein and whey fractions following a modified method in Jensen et al. [34]. Briefly, defatted milk was adjusted to pH 4.6 with 1 M acetic acid, incubated at 4°C for 10 min, and then neutralized with 1 M sodium acetate. After centrifugation (1500*g*, 10 min, 5°C), the supernatant (whey) was filtered through a 0.45 µm filter to remove residual casein micelles.

To assess hydrolyzed casein, we compared bovine casein sodium salt (Merck, USA, Cat. No. C8654) with casein peptone (Serva, Germany, Cat. No. 48600.04), both in concentration corresponding to casein concentration in milk (2.56 g/L). For hydrolyzed whey, whey protein (obtained from milk and diluted to its original concentration in milk) was treated with QIAGEN protease (Cat. No. 19157) (5 μL/10 mL whey) at 55°C for 60 min, then the enzyme was inactivated at 70°C for 30 min. Oleic acid (ROTH, Germany, Cat. No. 48600.04) and triolein (Merck, USA, Cat. No. Y0001113) were each diluted in water to 1.7 g/L, reflecting half the oleic acid concentration found in milk (3.4 g/L) [35].

### 2.3 Biorelevant Media

To simulate GI conditions, biorelevant media fasted state simulated gastric fluid (FaSSG), FaSSIF (fasted state simulated intestinal fluid) and fed state simulated intestinal fluid (FeSSIF) were obtained from Biorelevant.com (London, UK). Biorelevant media containing pH buffer and 3F powder (bile salts and lecithin) were prepared as instructed by the manufacturer. For simulations of fed gastric pH, FaSSG was adjusted to pH values of 3.0, 4.5, and 6.0. To assess the effect of milk and its fractions, each solution was mixed with milk or milk fractions at a 1:1 volume ratio and its pH adjusted to 1.6, 3.0, 4.5, and 6.0 if necessary.

### 2.4 Viral Stability Assays

To assess viral stability, TBEV (1 × 10^8^ PFU/mL) was diluted 1:10 in PBS and then mixed with each test solution at a 1:9 ratio. Samples were incubated in a shaking heat block (37°C, 270 rpm). At time points 1, 10, 30, 60, and 120 min, 30 μL aliquots were collected, immediately diluted 1:10 in 10% FBS in PBS (to neutralize acidic pH), and subjected to plaque assay.

For long‐term stability experiments, TBEV (1 × 10^8^ PFU/mL) was diluted 1:25 in PBS and then combined with test media 1:9. Samples were incubated at 8°C, and aliquots were collected on Days 0, 1, 2, 3, 5, and 7. Each aliquot (25 μL) was transferred into fresh DMEM (225 μL) and stored at −80°C until all time points were collected for titration.

### 2.5 Viral Affinity to Milk Fractions

To evaluate the potential binding affinity of TBEV to milk components, the virus (1 × 10^8^ PFU/mL) diluted 1:15 in PBS was mixed with whole milk in ratio 1:20. After centrifugation (14 300*g*, 15 min), 20 μL of each fraction (milk fat, skim milk, and pellet) was subjected to plaque assay. A control sample (whole milk with virus, not centrifuged) was processed identically.

### 2.6 Plaque Assay

Plaque assays were performed using A549 cells as described previously [36, 37]. Briefly, serial 10‐fold dilutions of viral suspension were prepared in 24‐well plates and cells (1 × 10^5^ cells/well) were added directly to the viral suspension in each well. After a 3–4‐h adsorption period at 37°C, cells were overlaid with DMEM containing 1.5% carboxymethylcellulose and incubated for 4 days. Then, the cell monolayers were washed with PBS and stained with naphthalene black. Titers were expressed as plaque‐forming units per milliliter (PFU/mL).

### 2.7 ELISA

The presence of specific anti‐TBEV antibodies in sheep, goat, and locally produced cow milk was assessed using ELISA IMMUNOZYM FSME (TBEV) IgG All Species Kit (Progen, Cat. No. 7701075). The ELISA kit was used according to manufacturer's instructions. Samples with antibody concentrations below 63 VIEU/mL were considered negative.

### 2.8 Statistical Analysis

All experiments were performed in hexaplicates (typically two independent experiments in biological triplicates) unless stated otherwise. Statistical comparisons of viral titers were performed using the nonparametric Mann–Whitney *U* test. Analyses were conducted using GraphPad Prism v7.0 (GraphPad Software, USA). A *p* value below 0.05 indicated statistical significance. The graphs show mean values; error bars denote standard error, and the dashed line marks plaque assay detection limits. We used the Kruskal–Wallis test to compare milk fractions after fractionalization.

## 3 Results

### 3.1 Milk Supports TBEV Stability at Low Temperature, but Reduces Viability During Short‐Term Incubation at Body Temperature

**Figure 1:**
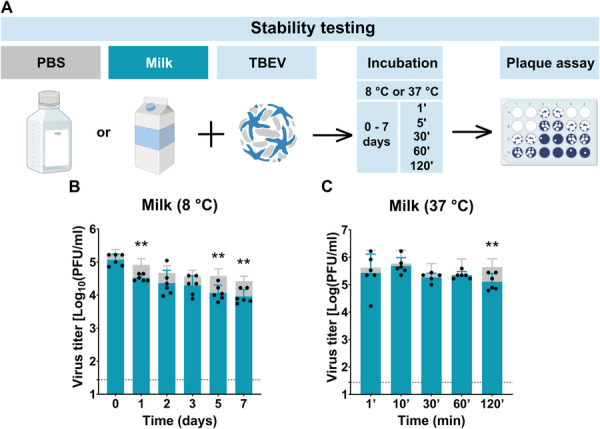
Stability of TBEV in milk and PBS. (A) Schematic overview of the experimental design: TBEV was mixed with either milk or PBS and incubated for 7 days or 1, 10, 30, 60, and 120 min at 8°C or 37°C. The mixtures were then neutralized using PBS with FBS, followed by plaque assay to determine viral titers. (B) TBEV titers in milk and PBS stored at 8°C, measured after 0, 1, 2, 3, 5, and 7 days. (C) TBEV titers in milk and PBS after incubation at 37°C for 1, 10, 30, 60, and 120 min with shaking (250 rpm). Gray columns represent titers in PBS in corresponding time of collection as a control in each experiment The graphs show mean values; error bars denote standard error, and the dashed line marks plaque assay detection limits. **p < 0.01.

To evaluate the role of milk in the persistence of TBEV under conditions relevant to alimentary transmission, we first investigated how the virus is affected by storage in milk over time (Figure 1A). Specifically, we compared the viral stability of TBEV in milk and PBS at refrigeration temperature (8°C) (Figure 1B) and at physiological temperature (37°C) (Figure 1C).

At 8°C, a statistically significant reduction in viral titer was observed in both milk and PBS after just 1 day of incubation (Supporting Information S2: Table 1) when compared to the initial titer. However, infective particles were still detected in both samples after 7 days. A small but statistically significant reduction in viral titer was consistently observed in milk compared to PBS. After 2 days, titers were 4.363 ± 0.387 log_10_ PFU/mL in milk versus 4.674 ± 0.210 in PBS (*p* = 0.0022). This trend continued at 5 days (4.070 ± 0.233 vs. 4.577 ± 0.221; *p* = 0.0087) and 7 days (3.960 ± 0.208 vs. 4.422 ± 0.150; *p* = 0.0065), indicating a gradual loss of infectivity in milk under cold storage conditions (Figure 1B).

To simulate short‐term exposure to body temperature, we incubated TBEV in milk and PBS at 37°C for 120 min (Figure 1C). The virus again exhibited a significantly greater decrease in titer in milk after 120 min (5.107 ± 0.300 log_10_ PFU/mL) than in PBS (5.642 ± 0.301; *p* = 0.0087), suggesting that components of milk may negatively affect TBEV stability under conditions resembling the upper GI environment.

Notably, a more pronounced destabilizing effect on TBEV was observed in goat milk, sheep milk, the raw cow milk, and its pasteurized variant used in the pasteurization experiments when incubated at 37°C. These differences in viral viability among different milk types are provided in the Supporting Information S2: Figure 1.

Prior to our experiments, samples of sheep, goat, and locally produced cow milk were tested by ELISA and found negative for TBEV‐specific antibodies. As the experimental results were consistent across different batches of milk, there was no evidence that batch variability influenced the outcomes of the short‐ or long‐term stability assessments or any other experiments.

### 3.2 Milk Accelerates TBEV Inactivation Under Simulated Gastric Conditions

**Figure 2:**
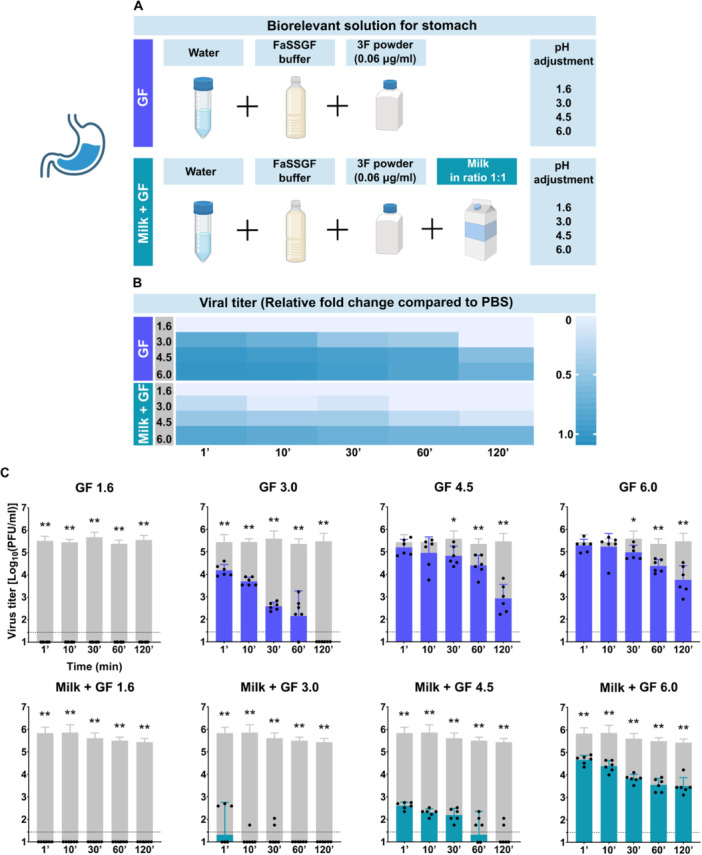
Stability of TBEV in biorelevant gastric fluid mimicking solutions. (A) Schema of preparation of simulated gastric fluid solutions. Each solution was prepared by mixing distilled water, FaSSGF buffer, and 3F powder. Milk was added where applicable. After mixing, the pH was adjusted to 1.6, 3.0, 4.5, or 6.0. Viral stability testing was performed as described previously, replacing milk or PBS with the test solutions. (B) Heatmap showing the relative fold change in TBEV titer compared to PBS in time. “GF” denotes gastric fluid; the number indicates the pH of the solution. (C) Time‐dependent change in viral titer during incubation. Gray columns represent titers in PBS in corresponding time of collection as a control in each experiment. All experiments were performed twice in triplicate. The graphs show mean values; error bars denote standard error, and the dashed line marks plaque assay detection limits. *p < 0.05; **p < 0.01.

To explore how gastric conditions influence the fate of TBEV during the early stages of digestion, we examined viral stability in biorelevant solutions simulating fasted and fed gastric environments at various pH levels (1.6, 3, 4.5, and 6). We also assessed whether milk modulates viral survival under these acidic conditions (Figure 2A).

In all tested gastric solutions, TBEV was rapidly inactivated compared to the PBS control. In the highly acidic fasted‐state gastric solution (pH 1.6), no infectious viral particles were detected even after just 1 min of incubation. In the pH 3 solution, simulating a fed stomach with lower acidity, a significant reduction in viral titer was observed after 1 min (4.176 ± 0.260 vs. 5.431 ± 0.331 log_10_ PFU/mL in PBS; *p* = 0.0022), and complete viral inactivation occurred within 120 min. Under moderately acidic conditions (pH 4.5 and 6), viral particles remained detectable after 120 min; however, the titers were still significantly lower than in PBS (pH 4.5: 2.926 ± 0.627 vs. 5.470 ± 0.346; *p* = 0.0022; pH 6: 3.754 ± 0.632 vs. 5.470 ± 0.346; *p* = 0.0022) (Figure 2B,C).

When milk was added to these gastric solutions (1:1 ratio), viral inactivation occurred more rapidly. In pH 1.6 solution containing milk, no infectious virus was detected after 1 min, identical to the milk‐free condition. However, the virus was cleared even faster in less acidic conditions. For instance, in pH 3 with milk, no virus was detected after just 60 min (vs. 120 min without milk). Notably, a significant reduction in titer was already present after 1 min in milk‐containing solutions: pH 3 (1.313 ± 1.439 vs. 5.834 ± 0.259 in PBS; *p* = 0.0022), pH 4.5 (2.608 ± 0.156 vs. 5.834 ± 0.259; *p* = 0.0022), and pH 6 (4.665 ± 0.197 vs. 5.834 ± 0.259; *p* = 0.0022) (Figure 2B,C).

When directly compared to solutions without milk, viral titers after 1 min were significantly lower in milk‐containing solutions at all pH levels tested: pH 3 (1.313 ± 1.439 vs. 4.176 ± 0.260; *p* = 0.0022), pH 4.5 (2.608 ± 0.156 vs. 5.197 ± 0.354; *p* = 0.0022), and pH 6 (4.665 ± 0.197 vs. 5.281 ± 0.275; *p* = 0.0022) (Figure 2B,C).

After 120 min, milk still exhibited a suppressive effect, most notably at pH 4.5 (0.632 ± 0.984 with milk vs. 2.926 ± 0.627 without; *p* = 0.0022). At pH 6, the difference was smaller and not statistically significant (3.501 ± 0.377 vs. 3.754 ± 0.632; *p* = 0.5152) (Figure 2B,C).

**Figure 3:**
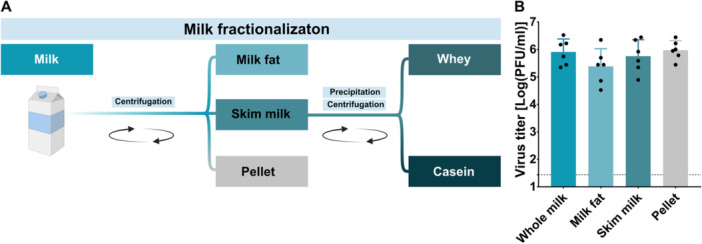
Fractionation of milk and affinity of TBEV to milk fractions. (A) Schematic of milk fractionation. Whole milk was centrifuged to separate the fat and skim milk layers. Casein was precipitated from skim milk using CH_3_COOH and removed by centrifugation. The resulting supernatant (whey) was filtered to eliminate residual casein. (B) TBEV titer in milk fractions following centrifugation. The graphs show mean values; error bars denote standard error, and the dashed line marks plaque assay detection limits.

Similar trend confirming negative effect of milk in the stomach was observed in the solutions with 1:3 and 1:7 ratio of milk to gastric fluid mimicking solution (Supporting Information S2: Figure 2) and in solutions with goat milk, sheep milk, and cow milk from local producer both before and after pasteurization (Supporting Information S2: Figure 3).

Together, these results demonstrate that the gastric environment is highly unfavorable for TBEV survival, and surprisingly, the presence of milk further enhances viral inactivation across a range of gastric pH values. This suggests that milk does not protect the virus in the stomach and may even facilitate its degradation under acidic conditions.

### 3.3 All Milk Fractions Contribute to TBEV Inactivation in Simulated Gastric Conditions

To assess viral affinity to individual components, TBEV was mixed with milk and samples were subsequently centrifuged. Comparison of viral titers in the separated fractions (milk fat, skim milk, and pellet) revealed no significant differences (*p* = 0.3272), indicating no preferential association of the virus with any particular milk component (Figure 3B).

To investigate the role of different milk components in the inactivation of TBEV in gastric‐like environments, milk was fractionated into fat and skim milk by centrifugation. The skim milk was further separated into whey and casein fractions via acetic acid (CH_3_COOH) precipitation and centrifugation (Figure 3A). TBEV was added to each fraction and subsequently cultivated for 2 h at 37°C.

**Figure 4:**
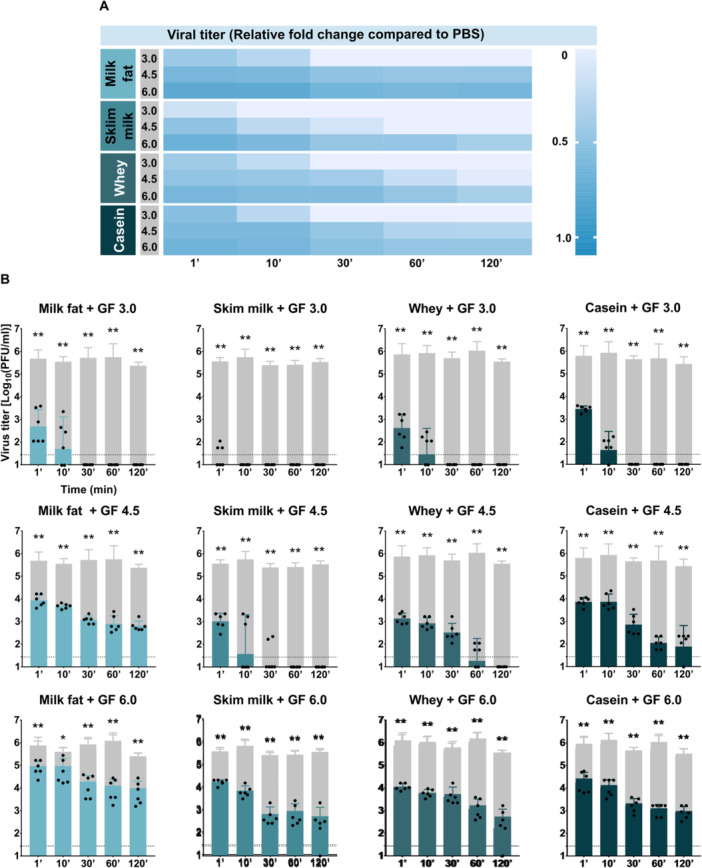
Effect of milk fractions on TBEV stability in simulated gastric fluid. (A) Heatmap showing the relative fold change in TBEV titer compared to PBS in time. “GF” denotes gastric fluid; the number indicates the pH of the solution. (B) Temporal changes in viral titer during incubation in each milk fraction. Gray columns represent titers in PBS in corresponding time of collection as a control in each experiment. All experiments were performed twice in triplicate. The graphs show mean values; error bars denote standard error, and the dashed line marks plaque assay detection limits. *p < 0.05; **p < 0.01.

When the antiviral activity of the milk fractions was evaluated across different pH conditions (3.0, 4.5, and 6.0), all tested fractions (fat, skim milk, whey, and casein) significantly reduced viral titers within 1 min of exposure compared to PBS controls (*p* < 0.05). No infectious virus was detected after 30 min at pH 3.0 in any milk fraction; notably, in skim milk at pH 3.0, viral inactivation occurred within 10 min (Figure 4A,B).

At pH 4.5, complete inactivation was observed in skim milk after 60 min, while in other fractions the virus remained detectable up to 120 min, albeit with significantly reduced titers compared to PBS. After 120 min at pH 4.5, viral titers in milk fat (2.804 ± 0.217 log_10_ PFU/mL) were similar to PBS (2.926 ± 0.627; *p* =  0.8485), while titers in whey (0.291 ± 0.714; *p* = 0.0022) and casein (1.885 ± 0.931; *p *= 0.0498) were significantly lower (Figure 4A,B).

At pH 6.0, infectious virus remained detectable in all fractions after 120 min. However, titers in skim milk (2.188 ± 1.138; *p* = 0.0065), whey (2.217 ± 1.147; *p* = 0.0087), and casein (2.918 ± 0.280; *p* = 0.0195) were significantly reduced compared to PBS controls (3.754 ± 0.632). In contrast, titers in the milk fat fraction (3.772 ± 0.495) remained similar to controls (*p* = 0.7473) (Figure 4A,B). Samples treated with hydrolyzed casein and whey proteins showed reduced viral stability (see Supporting Information S2: Figure 4).

**Figure 5:**
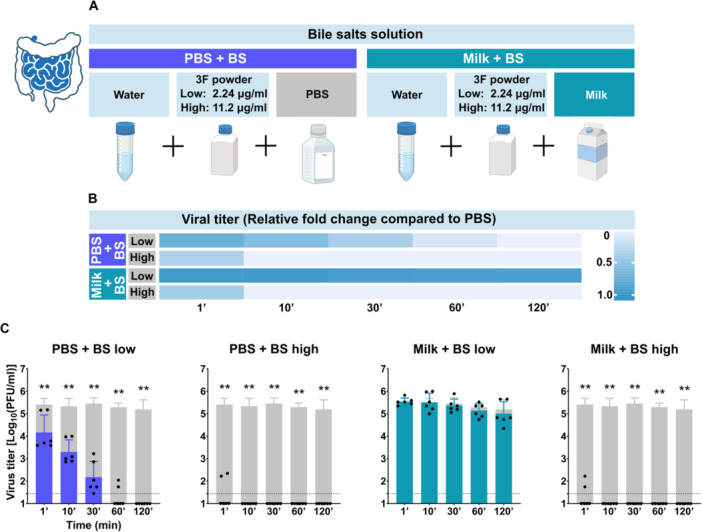
Effect of bile salts and milk on TBEV stability in simulated intestinal conditions. (A) Schematic representation of bile salts solution preparation. Solutions consisted of distilled water and 3F powder; milk was added where indicated. (B) Heatmap showing the relative fold change in TBEV titer compared to PBS in time. “BS” denotes bile salts; “BS low” corresponds to a bile salt concentration representative of the fasting intestine (2.24 μg/mL), and “BS high” reflects the fed intestinal concentration (11.2 μg/mL). (C) Temporal changes in viral titer during incubation in the presence of bile salt solutions with or without milk. Gray columns indicate TBEV titers in PBS in corresponding time of collection as a control in each experiment. All experiments were conducted twice in triplicate. The graphs show mean values; error bars denote standard error, and the dashed line marks plaque assay detection limits. **p < 0.01.

Additionally, treatment of TBEV with oleic acid at concentrations equivalent to those naturally present in milk fat [35], but in its fully dissociated free form (i.e., not incorporated in triacylglycerols), resulted in complete viral inactivation within 1 min of exposure, when the viral particles were treated with triolein to compare with dissociated oleic acid, the virus remained stable (Supporting Information S2: Figure 5).

### 3.4 Milk Protects TBEV Against Bile Salt–Mediated Inactivation

**Figure 6:**
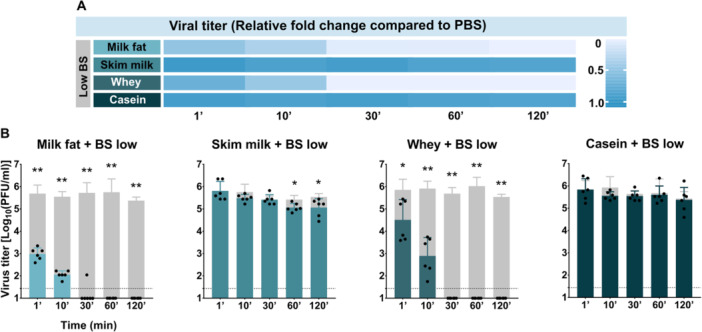
Protective effect of milk fractions against bile salt–mediated destabilization of TBEV. (A) Heatmap showing the relative fold change in TBEV titer compared to PBS in time. “BS” indicates bile salts; “BS low” represents the concentration found in the fasting intestine (2.24 μg/mL), and “BS high” corresponds to the fed state concentration (11.2 μg/mL). (B) Temporal progression of viral titer during incubation in bile salt solutions supplemented with different milk fractions. Gray columns represent titers in PBS in corresponding time of collection as a control in each experiment. All experiments were conducted twice in triplicate. The graphs show mean values; error bars denote standard error, and the dashed line marks plaque assay detection limits. *p < 0.05; **p < 0.01.

To assess the effect of intestinal conditions on TBEV stability, we replaced full simulated intestinal fluid with 3F powder—c—at physiologically relevant concentrations for fasting (2.24 μg/mL) and fed (11.2 μg/mL) states (Figure 5A). These were dissolved in PBS at a 1:1 ratio. Full simulated intestinal fluid would contain also FaSSIF and FeSSIF solution together with 3F powder, slightly adjusting pH of the biorelevant media. This substitution was based on comparable inactivation patterns with media containing only 3F powder or full simulated intestinal fluid (Supporting Information S2: Figure 6) and our prior detailed analysis of pH effects in gastric conditions.

In the high‐concentration bile salt solution (fed‐state equivalent), viral titers dropped significantly after 1 min in both PBS (0.662 ± 1.036 log_10_ PFU/mL) and milk (01.762 ± 1.180) compared to PBS control (5.408 ± 0.278; *p* = 0.0022 for both). No infectious virus was detectable after 10 min in either condition (Figure 5B,C).

In the low‐concentration bile salt solution (fasted‐state equivalent), no virus was detected in the PBS mixture after 120 min. A significant reduction was already observed after 1 min (4.167 ± 0.779 vs. 5.408 ± 0.278; *p* = 0.0238). However, when the virus was exposed to the same concentration of bile salts in milk, it remained stable. Viral titers after 120 min (5.006 ± 0.528) were not significantly different from the control without bile salts (5.195 ± 0.422; *p* = 0.6234), indicating a protective effect of milk against bile salt–mediated inactivation (Figure 5B,C).

Similar trend confirming positive effect of milk in the intestine was observed in the solutions with two and three times higher concentration of bile salts (Supporting Information S2: Figure 7) and in solutions with goat milk, sheep milk, and cow milk from local producer both before and after pasteurization (Supporting Information S2: Figure 8).

### 3.5 Casein Confers Protection Against Bile Salt–Mediated Inactivation

To identify the milk component responsible for protection against bile salts, we tested individual milk fractions—milk fat, skim milk, whey, and casein—each mixed with low‐concentration bile salts (fasted‐state equivalent).

In the milk fat fraction, viral titers were significantly reduced after 1 min (2.982 ± 0.272 vs. 5.690 ± 0.384; *p* = 0.0022), and no virus was detectable after 120 min. Similarly, in the whey fraction, titers decreased significantly after 1 min (4.512 ± 0.910 vs. 5.875 ± 0.475 log_10_ PFU/mL; *p* = 0.0430), with complete viral inactivation observed by 30 min (Figure 6A,B).

In contrast, skim milk showed moderate protective capacity. A statistically significant reduction in viral titer compared to PBS was observed at 60 min (5.065 ± 0.188 vs. 5.418 ± 0.187; *p* = 0.0195) and 120 min (5.063 ± 0.398 vs. 5.536 ± 0.150; *p* = 0.0152), though the absolute differences were small (Figure 5A,B).

Importantly, the casein fraction provided the highest level of protection. Viral titers remained stable over 120 min, with no statistically significant difference from PBS control (5.374 ± 0.559 vs. 5.804 ± 0.312; *p* = 0.1515), indicating that casein is likely the key component mediating the protective effect of milk against bile salts (Figure 5A,B). Viral stability disappeared when bile salts solutions were combined with hydrolyzed casein (Supporting Information S2: Figure 9).

## 4 Discussion

TBEV is a neurotropic flavivirus typically transmitted via tick bites but can also be acquired alimentarily, most commonly through consumption of unpasteurized dairy products [4, 5, 10, 14]. While this route of infection is well‐documented, the mechanisms by which TBEV survives the hostile conditions of the GI tract remain poorly understood. In this study, we investigated the stability of TBEV in milk and its individual components under conditions simulating the gastric and intestinal environments, with the aim of identifying the factors contributing to viral survival or inactivation along the digestive route.

Our results demonstrate that TBEV remains relatively stable in milk under cold storage, with a moderate reduction in viral titer observed over 7 days at 8°C. This suggests that a considerable portion of viral particles remains infectious even after prolonged refrigeration. These findings are consistent with previous reports of TBEV stability at low temperatures in dairy products [19]. Additionally, when incubated at 37°C to simulate body temperature, no statistically significant decrease in viral titer was observed over 120 min, indicating that TBEV remains stable in milk for at least 2 h under physiological conditions.

Despite historical reports suggesting TBEV can remain infectious in the stomach for up to 2 h at low pH (pH 1.5–1.8) [16], our findings suggest rapid inactivation at such acidic conditions. At pH 1.5, no viral particles were detected after even 1 min, confirming the virus's high sensitivity to very low pH. This is in agreement with recent findings [17], though we observed a higher tolerance at pH 3, with viable virus still detectable after 1 h. These differences may stem from experimental conditions or strain variation.

When milk was added to low‐pH environments mimicking gastric conditions (pH 3, 4.5, and 6), a further reduction in viral stability was observed across all tested pH values. This suggests that milk components exacerbate TBEV destabilization under acidic conditions. Given that postprandial gastric pH drops from ~6 to ~3 within the first hour and to ~1.6 within 2 h [21], it is unlikely that TBEV remains infectious in the stomach for prolonged periods. Instead, we hypothesize that alimentary infection results from the rapid passage of infectious viral particles through the stomach before they are inactivated, consistent with evidence that portions of liquid meals can exit the stomach within minutes [38, 39].

To identify which milk components contribute to viral destabilization under acidic conditions, we fractionated milk and tested individual components. Initial separation and plaque assay analysis showed no preferential affinity of TBEV for any particular milk fraction (fat, skim milk, or pellet), and virus could be detected in all. This confirmed the suitability of the fractionation approach for assessing antiviral effects.

When testing viral stability in each milk fraction under acidic conditions, we found that the fat fraction had a destabilizing effect at pH 3 but not at pH 4.5 or 6. We attribute this effect to the release of free fatty acids from triacylglycerols under acidic conditions, as these free fatty acids—particularly oleic acid—have been reported to disrupt viral membranes [40, 41]. Our findings demonstrate that dissociated oleic acid, at physiological concentrations similar to those found in bovine milk [35], fully inactivated TBEV after just 1 min of exposure. In contrast, when oleic acid was present as part of triolein at comparable concentrations, TBEV exhibited stability. These results provide confirmation of our initial hypothesis. The skim milk fraction also exerted a destabilizing effect on TBEV across all tested pH conditions. Further separation revealed that both whey and casein contributed to this activity. Whey proteins, including lactoferrin, have well‐documented antiviral properties [42, 43, 44], while some studies have also described antiviral effects of casein [45, 46]. Notably, we did not observe significant antiviral effects in milk at neutral pH, suggesting that acidic conditions are required for these milk proteins to exert their activity against TBEV. Even mildly acidic pH (pH 6) was sufficient to trigger this effect. Both hydrolyzed casein and whey proteins showed destabilizing effects, indicating that antiviral activity may result from peptides in acidic conditions rather than the proteins themselves.

We next investigated TBEV stability in simulated intestinal conditions, focusing on the impact of bile salts. Exposure to bile salt concentrations mimicking fed‐state intestines led to rapid viral inactivation, with no virus detectable after 10 min. Even at lower concentrations corresponding to fasting conditions, TBEV was inactivated within 120 min. These findings align with previous studies reporting antiviral activity of bile salts [47, 48], although in contrast, bile salts may enhance replication in hepatotropic viruses [49, 50, 51].

When PBS was replaced with milk in the bile salt solution, a strong protective effect was observed at the lower bile salt concentration: TBEV remained stable for the full 120‐min incubation. This protective effect was not seen at the higher bile salt concentration. To determine the responsible milk component, we tested individual fractions. The fat fraction failed to protect the virus, whereas the skim milk fraction conferred partial protection. Further separation showed that intact casein best preserved viral stability, with no significant titer loss over 120 min. When replaced by hydrolyzed casein, this protective effect was eliminated, indicating the importance of casein's integrity. This aligns with prior findings that casein can bind bile salts [52, 53], offering a plausible mechanism for the observed protection.

In summary, our results suggest that while milk may initially protect TBEV in the intestine—likely through casein‐mediated sequestration of bile salts—it contributes to viral destabilization in the acidic environment of the stomach. The presence of membrane‐disrupting fatty acids and antiviral milk proteins (whey and casein) in acidic conditions accelerates viral inactivation. These findings help explain how alimentary infection may occur despite the harsh conditions of the digestive tract and identify specific milk components that modulate viral stability in a compartment‐specific manner.

## Author Contributions

Martin Machacek and Michaela Berankova planned and performed the experiments and wrote the draft of the manuscript. Jiri Salat and Daniel Ruzek supervised the work. Daniel Ruzek and Michaela Berankova conceived the study, supervised the study, and wrote the draft of the manuscript. All authors reviewed and discussed the results and reviewed and edited the manuscript.

## Conflicts of Interest

The authors declare no conflicts of interest.

## Supporting information

Supplementary Information – primary data.

Supplementary Table and Figures.

## Data Availability

All data generated or analyzed during this study are included in this published article and its Supporting Information S1: Supporting Information.
