# Supplementary material for: Milk Modulates the Gastrointestinal Stability of Tick‐Borne Encephalitis Virus: Implications for Alimentary Transmission
Source: J Med Virol. 2025 Dec 24;98(1):e70778. doi: 10.1002/jmv.70778 (PMC12730492; doi:10.1002/jmv.70778)
Supplement: Supplementary file 2 — Supplementary Table and Figures. [file JMV-98-e70778-s002.pdf]

Supplementary Material

Milk Modulates the Gastrointestinal Stability of Tick-Borne Encephalitis Virus: Implications for Alimentary Transmission

Martin Machacek,[1,2] Michaela Berankova,[1,2,3] Jiri Salat,[1,2,3] Daniel Ruzek[1,2,3,✉]

(1) Department of Experimental Biology, Faculty of Science, Masaryk University, Kamenice 735, CZ-62500 Brno, Czechia.

(2) Laboratory of Emerging Viral Infections, Veterinary Research Institute, Hudcova 296, CZ-62100 Brno, Czechia.

(3) Laboratory of Arbovirology, Institute of Parasitology, Biology Centre of the Czech Academy of Sciences, Branisovska 31, CZ-37005 Ceske Budejovice, Czechia.

✉Author for Correspondence: ruzekd@paru.cas.cz

Supplementary Table 1 | TBEV Stability in Milk Over Time.

Sample name	Sample titer log[(PFU/ml)]						Average	SD	M-W test sample in given time against sample after 0 days (p-value)	Statistical significance
milk 8 °C; 0 days	5,222	4,891	5,000	4,859	5,222	5,222	5,069	0,174	*1,0000*	–
milk 8 °C; 1 days	4,444	4,648	4,444	4,444	4,523	4,648	4,525	0,100	*0,0022*	**
milk 8 °C; 2 days	4,222	5,046	4,523	4,347	3,975	4,067	4,363	0,387	*0,0130*	*
milk 8 °C; 3 days	3,891	4,590	4,023	4,590	4,347	4,347	4,298	0,289	*0,0022*	**
milk 8 °C; 5 days	3,921	4,222	4,000	4,046	3,786	4,444	4,070	0,233	*0,0022*	**
milk 8 °C; 7 days	3,891	3,824	4,222	4,222	3,745	3,859	3,960	0,208	*0,0022*	**
PBS 8 °C; 0 days	5,067	5,222	5,444	5,347	5,222	5,067	5,228	0,150	*1,0000*	–
PBS 8 °C; 1 days	5,222	5,023	4,921	4,824	4,824	4,699	4,919	0,184	*0,0130*	*
PBS 8 °C; 2 days	4,444	4,786	4,859	4,444	4,590	4,921	4,674	0,210	*0,0022*	**
PBS 8 °C; 3 days	4,347	4,444	4,891	4,523	4,523	4,648	4,562	0,189	*0,0022*	**
PBS 8 °C; 5 days	4,222	4,590	4,444	4,648	4,859	4,699	4,577	0,221	*0,0022*	**
PBS 8 °C; 7 days	4,222	4,347	4,648	4,347	4,523	4,444	4,422	0,150	*0,0022*	**

Sample name	Sample titer log[(PFU/ml)]						Average	SD	M-W test sample in given time against sample after 1’ (p-value)	Statistical significance
milk 37 °C; 1’	5,347	5,523	5,347	4,222	6,222	5,921	5,430	0,686	*1,000*	–
milk 37 °C; 10’	6,222	5,347	5,590	5,523	5,786	5,648	5,686	0,300	*0,509*	–
milk 37 °C; 30’	5,000	5,347	5,222	5,347	5,444	5,222	5,263	0,154	*0,152*	–
milk 37 °C; 60’	5,347	5,347	5,222	5,222	5,347	5,590	5,346	0,134	*0,266*	–
milk 37 °C; 120’	4,786	4,859	5,444	5,444	5,222	4,891	5,107	0,300	*0,223*	–
PBS 37 °C; 1’	5,523	5,444	5,347	6,347	6,347	4,745	5,625	0,622	*1,000*	–
PBS 37 °C; 10’	5,347	5,452	5,530	5,523	6,347	6,444	5,774	0,487	*0,509*	–
PBS 37 °C; 30’	5,590	5,786	5,648	4,949	5,523	4,975	5,412	0,359	*0,998*	–
PBS 37 °C; 60’	5,699	5,699	5,222	5,699	5,590	4,222	5,355	0,585	*0,913*	–
PBS 37 °C; 120’	5,590	5,648	5,523	5,347	6,222	5,523	5,642	0,301	*1,000*	–

Upper table shows TBEV titers in milk and PBS during 7 days long cultivation at 8 °C and statistical comparison of the titers in the samples in given time and the titer at the start of the cultivation. Lower table shows TBEV titers in milk and PBS during 120 minutes days long cultivation at 37 °C and statistical comparison of the titers in the samples in given time and the titer after one minute of cultivation. –, $p>0.05$; *, $p < 0.05$; **, $p < 0.01$.

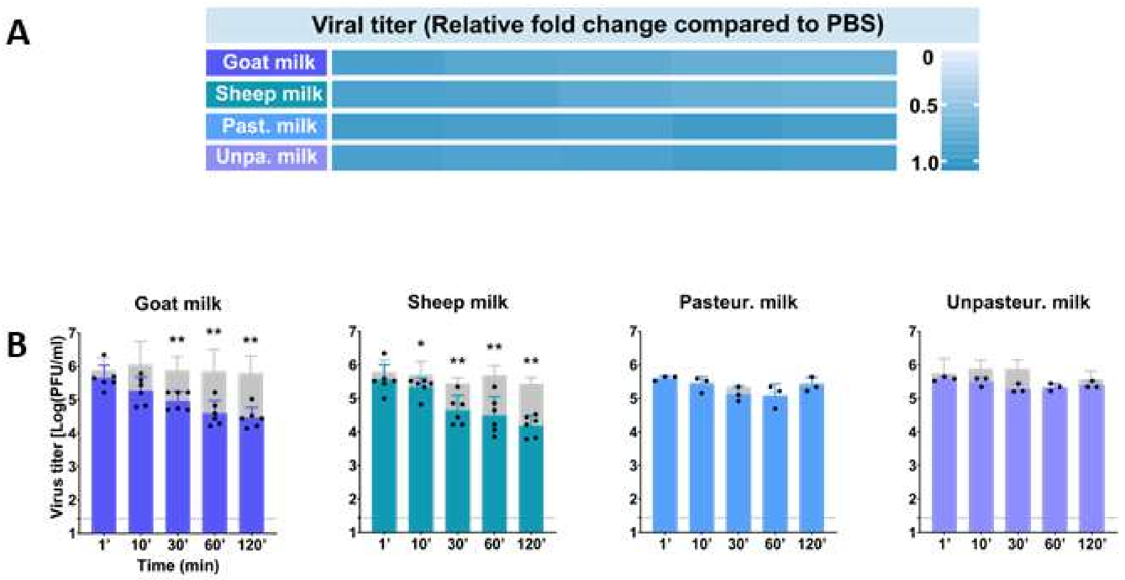

Supplementary Figure 1 | Stability of TBEV in different types of milk. (A) Heatmap showing the relative fold change in TBEV titer over time compared to PBS. "Pasteur. milk" refers to pasteurized cow milk from a local producer, while "Unpasteur. milk" denotes the same batch of milk before pasteurization. (B) Time-dependent changes in viral titer during incubation. Grey bars represent TBEV titers in PBS at the corresponding time points, serving as controls in each experiment. Experiments with sheep and goat milk were performed twice in triplicate; experiments with locally sourced cow milk were performed in triplicate. *, $p < 0.05$; **, $p < 0.01$.

A

B

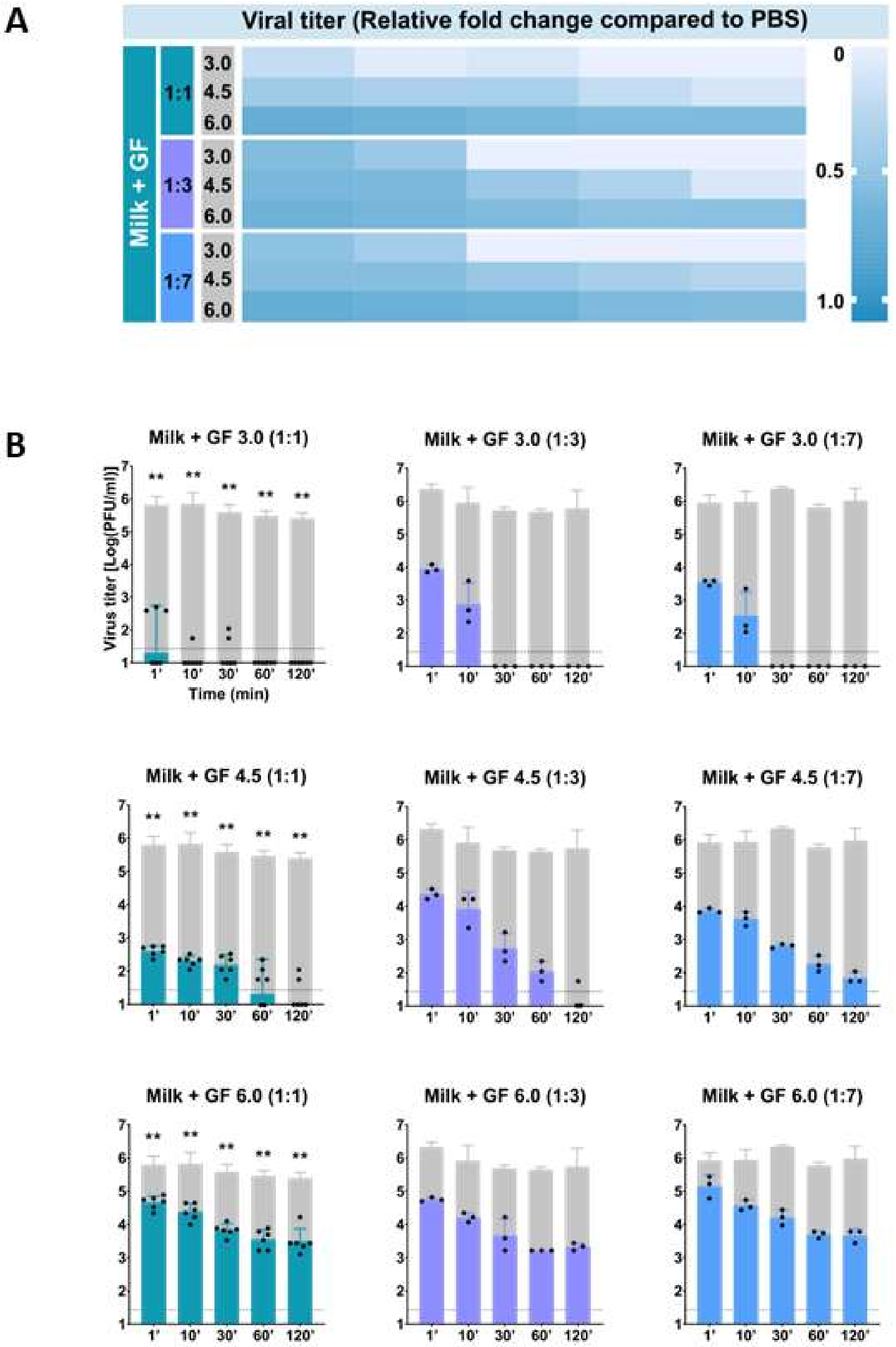

Supplementary Figure 2 | Effect of milk fractions on TBEV stability in biorelevant gastric fluid mimicking solutions, depending on the milk-to-gastric fluid ratio. (A) Heatmap showing the relative fold change in TBEV titer over time compared to PBS. "GF" denotes gastric fluid, with the accompanying number indicating the pH of the solution. (B) Time-dependent changes in viral titer during incubation in each milk fraction. Grey columns represent TBEV titers in PBS at the corresponding time points, serving as controls in each experiment. Experiments with the 1:1 ratio were performed twice in triplicate, while experiments with 1:3 and 1:7 ratios were performed in triplicate. **, $p < 0.01$.

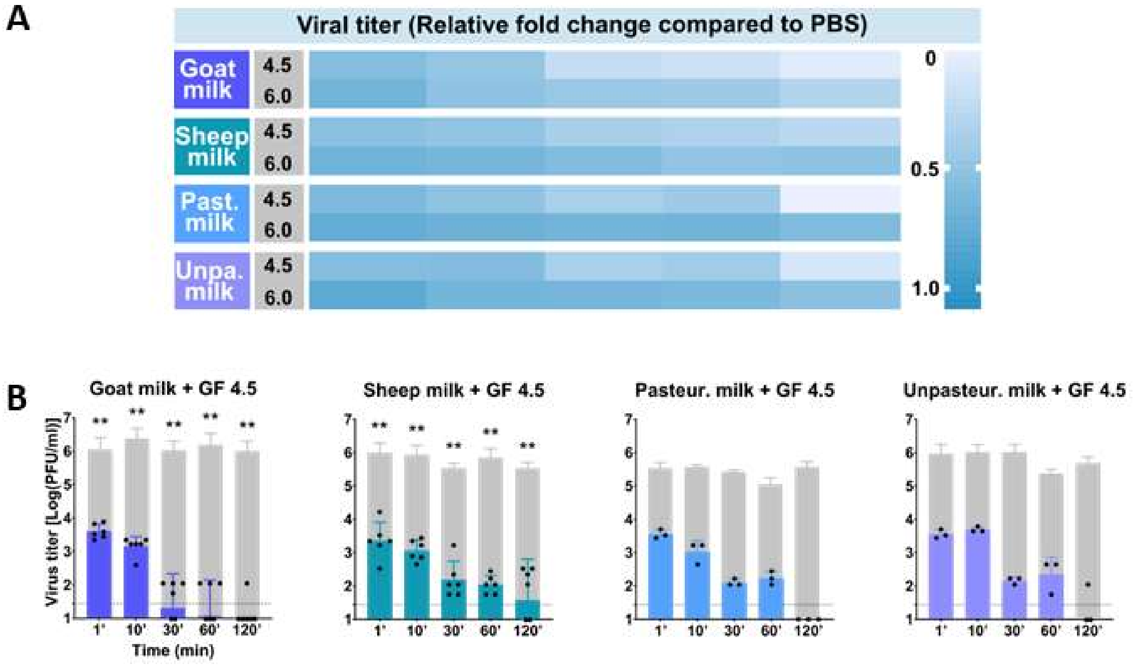

Supplement Figure 3 | Stability of TBEV in biorelevant gastric fluid mimicking solutions in different kinds of milk. (A) Heatmap showing the relative fold change in TBEV titer compared to PBS in time. “GF” denotes gastric fluid; the number indicates the pH of the solution. “Pasteur. milk” refers to pasteurized cow milk from a local producer, while “Unpasteur. milk” denotes the same batch of milk before pasteurization. (B) Time-dependent change in viral titer during incubation. Grey bars represent titers in PBS in corresponding time of collection as a control in each experiment. Experiments with sheep and goat milk were performed twice in triplicate, experiment with locally supplemented cow milk were performed in triplicate. **, $p < 0.01$.

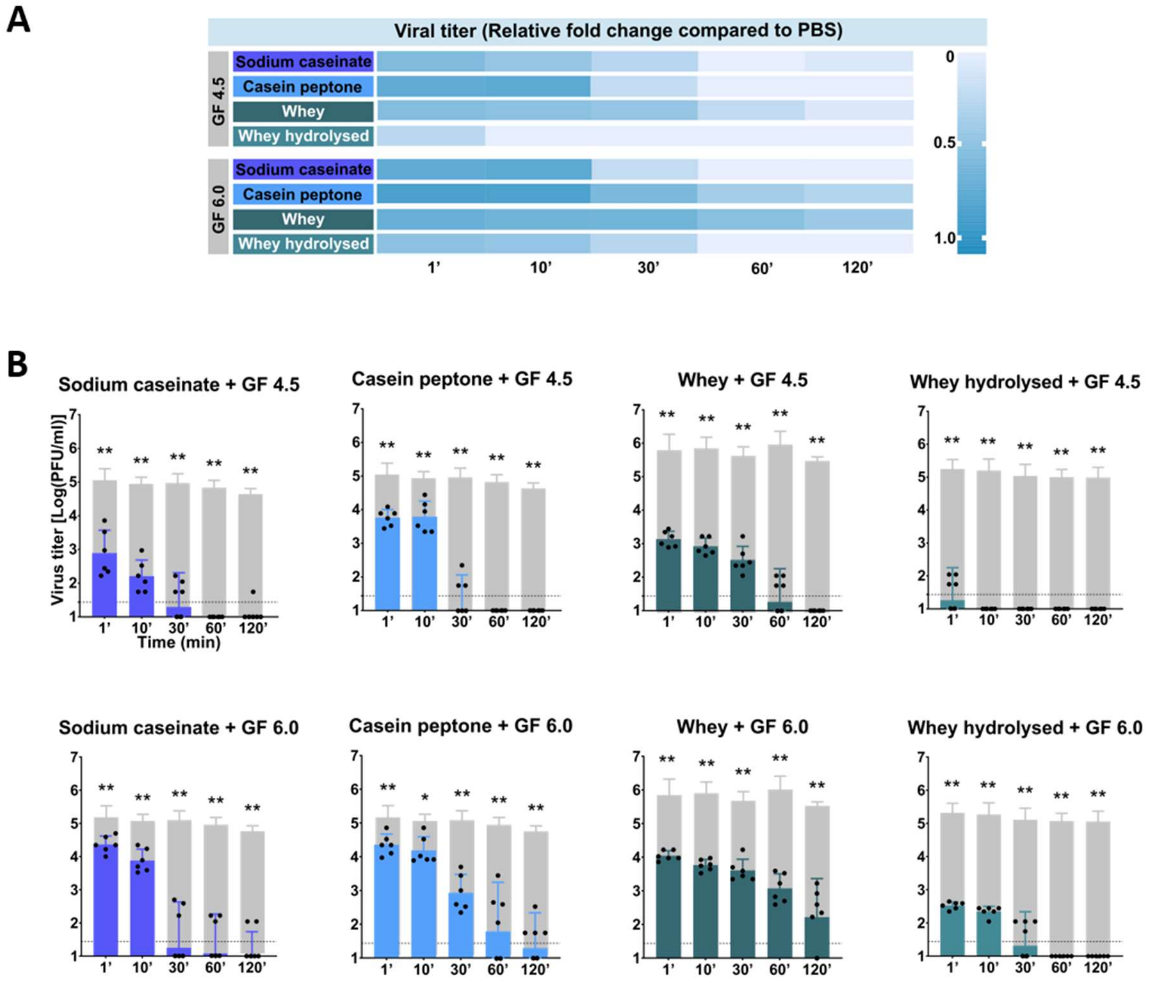

Supplementary Figure 4 | Stability of TBEV in biorelevant gastric fluid mimicking solutions in hydrolysed whey and casein. (A) Heatmap showing the relative fold change in TBEV titer over time compared to PBS. "GF" denotes gastric fluid; the number indicates the pH of the solution. Both sodium caseinate and casein peptone samples were tested in concentration corresponding to the naturally occurring concentration of casein in milk (2.56 g/l). Both samples for testing of whey were isolated directly from milk, hydrolysed whey was digested with QIAGEN Protease (B) Time-dependent changes in viral titer during incubation. Grey bars represent TBEV titers in PBS at the corresponding time points, serving as controls in each experiment. Experiments were performed twice in triplicate. *, $p < 0.05$; **, $p < 0.01$.

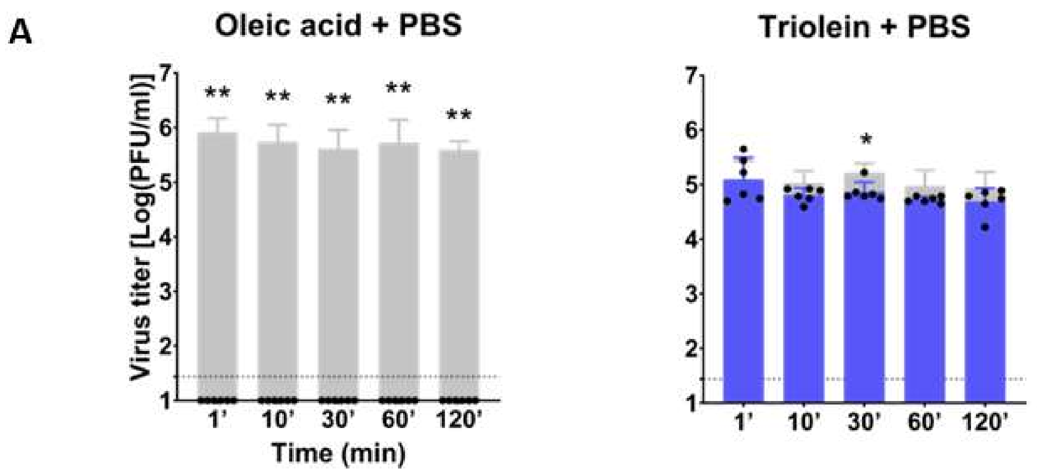

Supplementary Figure 5 | Stability of TBEV in oleic acid and triolein. (A) Time-dependent changes in viral titer during incubation. The concentration of oleic acid and triolein (3.4 g/l for both) corresponds to its natural concentration in milk. Grey bars indicate TBEV titers in PBS at the corresponding time points, serving as controls in the experiment. Experiments were performed in triplicate. *, $p < 0.05$; **, $p < 0.01$.

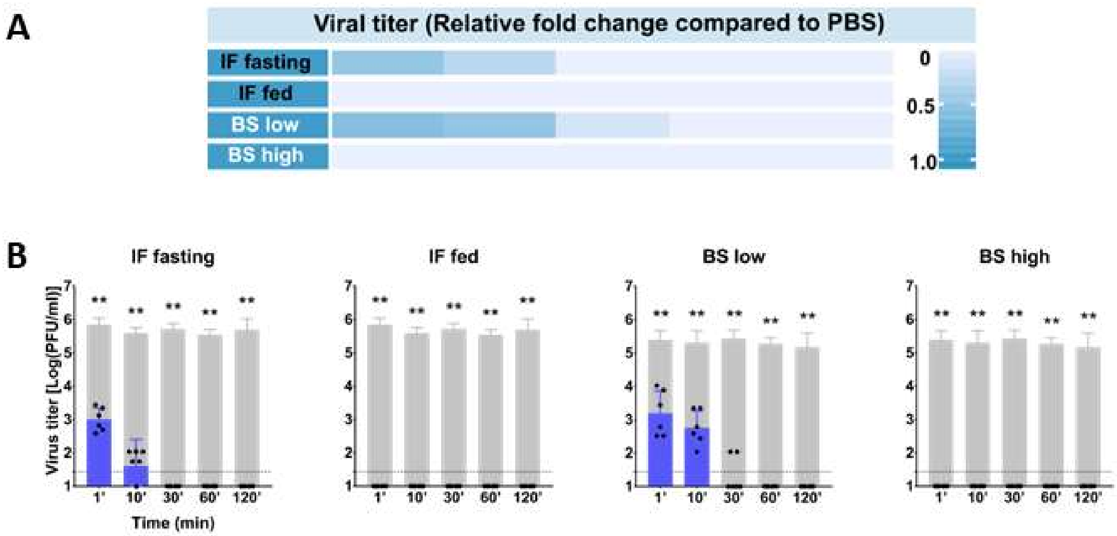

Supplementary Figure 6 | Stability of TBEV in biorelevant intestinal fluid mimicking solutions and in bile salts solutions. (A) Heatmap showing the relative fold change in TBEV titer over time compared to PBS. “IF” denotes intestinal fluid; “BS” denotes bile salts. “BS low” corresponds to a bile salt concentration representative of the fasting intestine (2.24 µg/mL), while “BS high” reflects the fed intestinal concentration (11.20 µg/mL). (B) Time-dependent changes in viral titer during incubation. Grey bars indicate TBEV titers in PBS at the corresponding time points, serving as controls in each experiment. Experiments were performed twice in triplicate. **, $p < 0.01$.

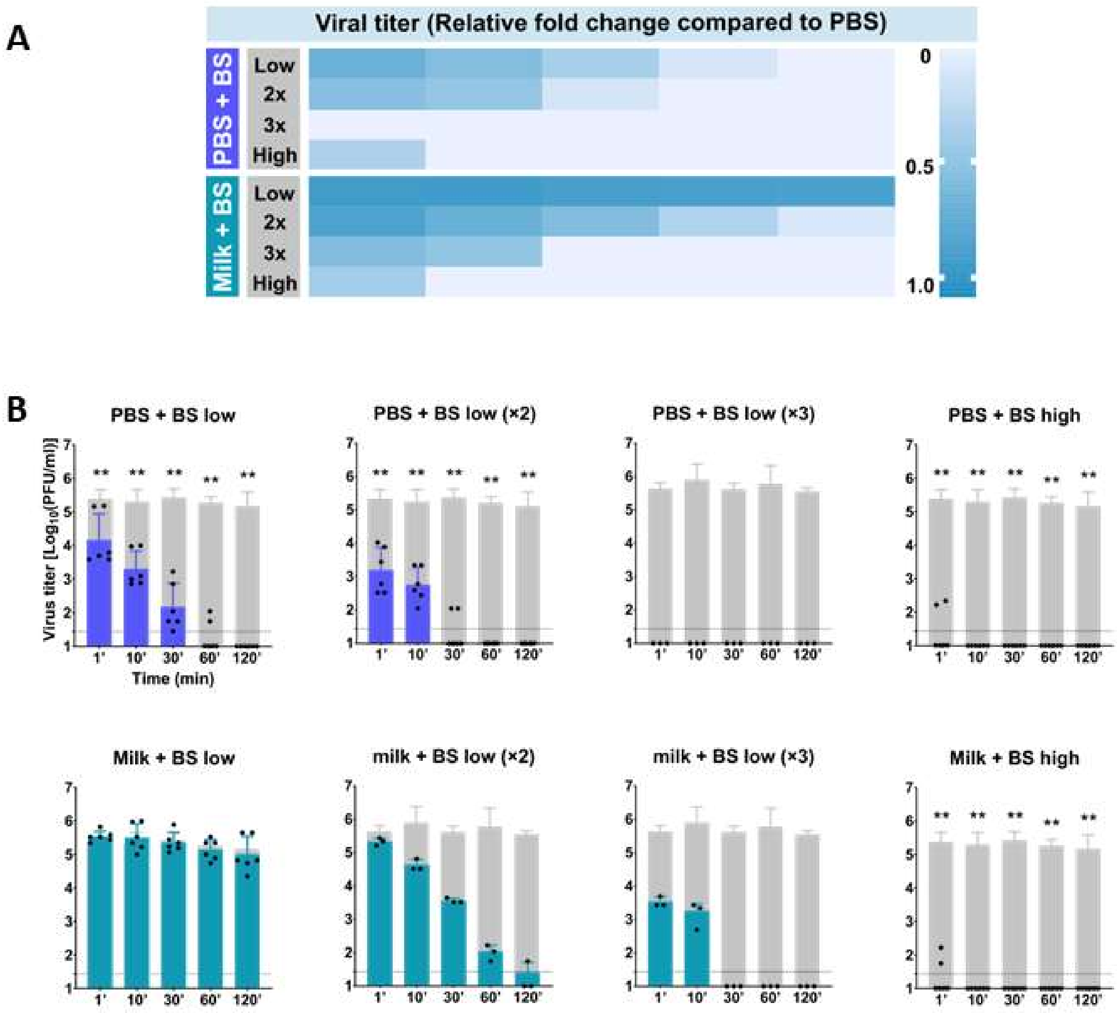

Supplementary Figure 7 | Stability of TBEV in bile salts solutions depending on bile salts concentration. (A) Heatmap showing the relative fold change in TBEV titer over time compared to PBS. "BS" denotes bile salts; "BS low" corresponds to a bile salt concentration representative of the fasting intestine (2.24 µg/mL), "BS low (×2)" to twice this concentration (4.48 µg/mL), and "BS low (×3)" to three times this concentration (6.72 µg/mL). "BS high" reflects the bile salt concentration typical of the fed intestine (11.20 µg/mL). (B) Time-dependent changes in viral titer during incubation. Grey bars represent TBEV titers in PBS at the corresponding time points, serving as controls in each experiment. Experiments with "BS low" and "BS high" were performed twice in triplicate; experiments with "BS low (×2)" and "BS low (×3)" were performed in triplicate. **, $p < 0.01$.

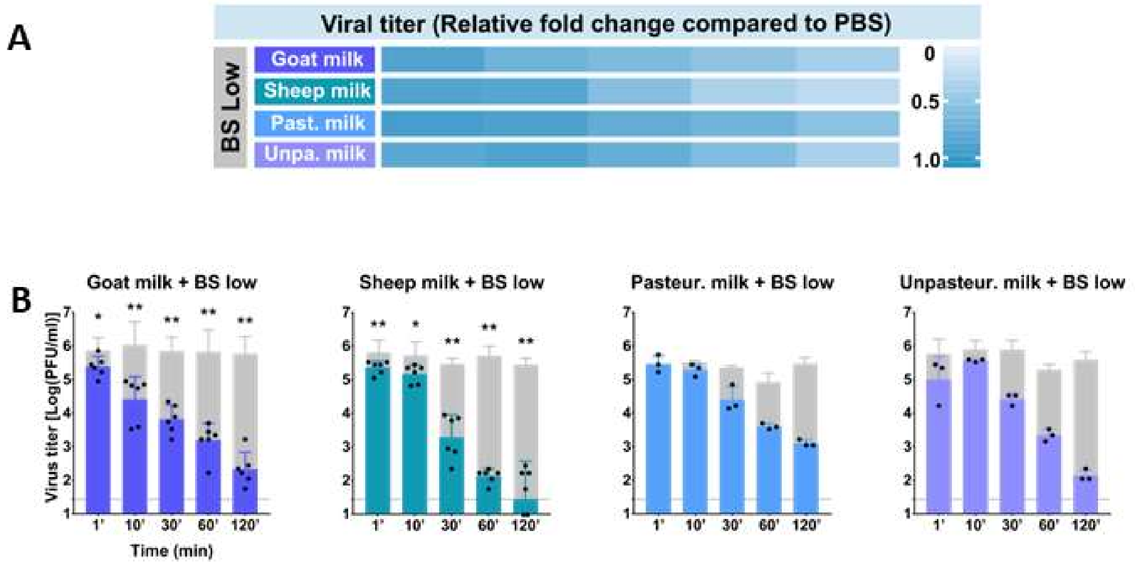

Supplementary Figure 8 | Stability of TBEV in bile salts solutions in different kinds of milk. (A) Heatmap showing the relative fold change in TBEV titer over time compared to PBS. "BS" denotes bile salts; "BS low" corresponds to a bile salt concentration representative of the fasting intestine (2.24 µg/mL), while "BS high" reflects the concentration in the fed intestine (11.20 µg/mL). "Pasteur. milk" refers to pasteurized cow milk from a local producer, and "Unpasteur. milk" denotes the same batch of milk before pasteurization. (B) Time-dependent changes in viral titer during incubation. Grey bars represent TBEV titers in PBS at the corresponding time points, serving as controls in each experiment. Experiments with sheep and goat milk were performed twice in triplicate, while experiments with locally sourced cow milk were performed in triplicate. *, $p < 0.05$; **, $p < 0.01$.

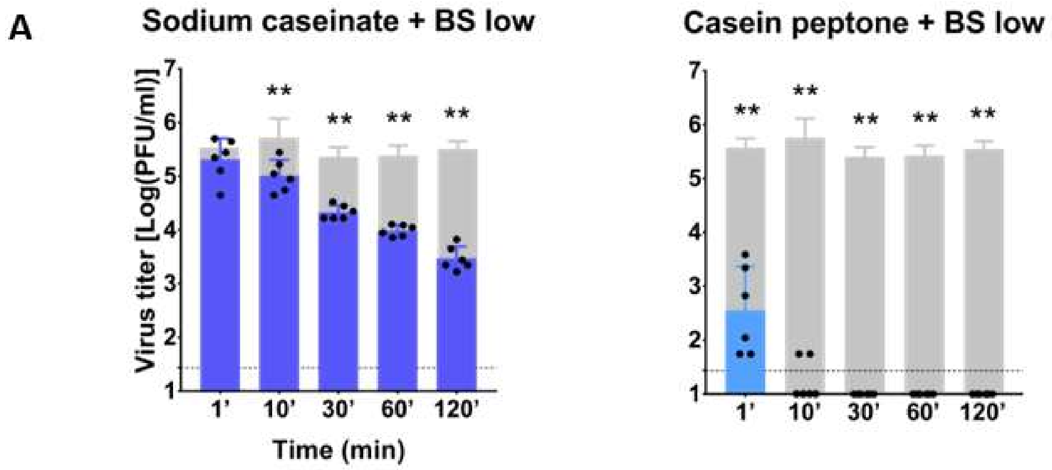

Supplementary Figure 9 | Stability of TBEV in in bile salts solutions in hydrolysed casein. (A) Time-dependent changes in viral titer during incubation. "BS" denotes bile salts. "BS low" corresponds to a bile salt concentration representative of the fasting intestine (2.24 µg/mL) Both sodium caseinate and casein peptone samples were tested in concentration corresponding to the naturally occurring concentration of casein in milk (2.56 g/l). Grey bars represent TBEV titers in PBS at the corresponding time points, serving as controls in each experiment. Experiments were performed twice in triplicate. *, p < 0.05; **, p < 0.01.
